# Underlying beneficial effects of Rhubarb on constipation-induced inflammation, disorder of gut microbiome and metabolism

**DOI:** 10.3389/fphar.2022.1048134

**Published:** 2022-12-05

**Authors:** Han Gao, Chengwei He, Rongxuan Hua, Chen Liang, Boya Wang, Yixuan Du, Shuzi Xin, Yuexin Guo, Lei Gao, Lucia Zhang, Hongwei Shang, Jingdong Xu

**Affiliations:** ^1^ Department of Physiology and Pathophysiology, School of Basic Medical Sciences, Capital Medical University, Beijing, China; ^2^ Department of Clinical Medicine, School of Basic Medical Sciences, Capital Medical University, Beijing, China; ^3^ Undergraduate Student of 2018 Eight Program of Clinical Medicine, Peking University People^'^s Hospital, Beijing, China; ^4^ Department of Oral Medicine, School of Basic Medical Sciences, Capital Medical University, Beijing, China; ^5^ Department of Biomedical Informatics, School of Biomedical Engineering, Capital Medical University, Beijing, China; ^6^ Class of 2025, Loomis Chaffee School, Windsor, CT, United States; ^7^ Experimental Center for Morphological Research Platform, School of Basic Medical Sciences, Capital Medical University, Beijing, China

**Keywords:** constipation, rhubarb extract, gut microbiome, polyamine, SCFA

## Abstract

**Background:** Constipation is a common syndrome and a worldwide healthy problem. Constipation patients are becoming younger, with a 29.6% overall prevalence in children, which has captured significant attention because of its epigenetic rejuvenation and recurrent episodes. Despite the usage of rhubarb extract to relieve constipation, novel targets and genes implicated in target-relevant pathways with remarkable functionalities should still be sought for.

**Materials and methods:** We established a reliable constipation model in C57B/6N male mice using intragastric administration diphenoxylate, and the eligible subjects received 600 mg/25 g rhubarb extract to alleviate constipation. Resultant constipation was morphological and genetically compared with the specimen from different groups.

**Results:** Constipation mice exhibited thicker muscle layers, higher levels of cytokines, including IL-17 and IL-23, and lower content of IL-22. Bacterial abundance and diversity varied tremendously. Notably, the alterations were reversed following rhubarb extract treatment. Additionally, Constipation also had a substantial impact on short-chain fatty acids (SCFAs), medium- and long-chain fatty acids (MLCFAs), and the expression of SCFA receptors, GPR41 and GPR43.

**Conclusion:** This thesis has provided insight that rhubarb extract promoted the flexibility of collagen fiber, reduced pro-inflammatory cytokines, enhanced anti-inflammatory cytokines, and maintained gut microflora balance with potential impacts on the fatty acid and polyamine metabolism.

## Graphical Abstract

**Figure:**
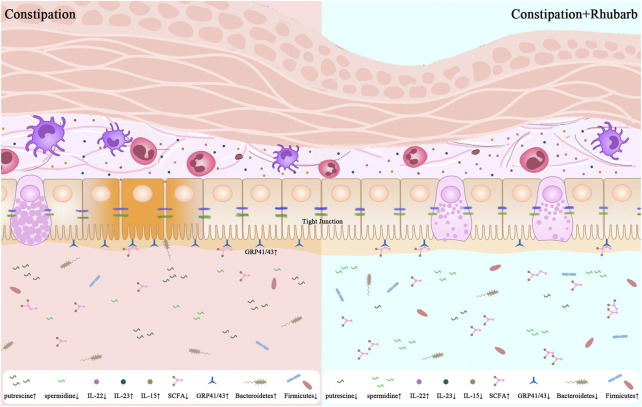
1. Constipation leads to the enhanced thickness of muscle layer and sedimentation of collagenous fiber. 2. Constipation promotes not only the pro-inflammtory cytokines secretion but also damage the integrity of intestinal barrier. 3. The present results are significant in at least two major respects in the alternations of the diversity of gut microbiome along with the fluctuation of the fatty acid and bioamine in constipation mice. 4. One unanticipated finding is that the rhubarb extract has the potential to reverse the alterations induced by constipation.

## 1 Introduction

Constipation is a prevalent clinical sign of gastrointestinal dysfunction with a 15% global occurrence rate. It is distinguished by difficult or infrequent passage or hardness of stool, and/or a sense of incomplete evacuation (Bharucha, 2007; Zhao and Yu, 2016). With the typical lifestyle of high consumption of sugar and fat, the prevalence of constipation is estimated as 20% or higher, which has serious consequences for the quality of people’s lives regardless of age and gender (Devanarayana and Rajindrajith, 2010). Rhubarb is an essential traditional Chinese medicinal herb applied to clinical practice for relieving constipation. A majority of current research on constipation has focused on motility enhancement. Motility of the gastrointestinal tract is an imprecise term embracing several measurable phenomena, including enteric contractile activity, gut wall biomechanical functions, and intraluminal flow responsible for the propulsion of gut contents (Dimidi et al., 2017). To uncover whether rhubarb exerted influence on gut motility, we evaluated correlations between the muscle alterations and collagen fibers density to demonstrate the sensitivity enhancements of colonic contraction.

The vast majority of gut microbes represent a highly complex microenvironment assembly of an estimated 10–100 trillion symbiotic bacteria per individual, all of which are present in intimate contact with the host and correlate with health and disease (Ventura et al., 2009; Hamer et al., 2012). A number of studies provide strong evidence that the microbes and their hosts share a wide range of resources required to maintain physiological requirements (Donohoe et al., 2011; Kuwahara, 2014). More importantly, the gut microbiota actively creates a great deal of immune regulatory metabolites (Jarchum and Pamer, 2011).

Short-chain fatty acids (SCFAs), major end products of gut microbial fermentation and an energy source of epithelial cells, take part in regulating the gut immune response (Ventura et al., 2009; Macia et al., 2012). They promote mucin production and the expression of antimicrobial peptides (Kles and Chang, 2006). SCFA receptors include G-protein-coupled receptors (GPR) such as GPR41 and GPR43, which allow SCFA to activate different cells such as epithelial cells, adipocytes, and phagocytes while also regulating other physiological activities (Tazoe et al., 2008). Additionally, data suggests that SCFA and its receptors contribute to acute inflammatory responses in the gut (Kim et al., 2013).

Biogenic amines are conventionally produced *via* microbial fermentation of undigested amino acids by deamination, deamination-decarboxylation, or carboxylation (Walker et al., 2005; Holmes et al., 2017). To the best of our understanding, the most polyamines in this region of the colon are produced depending on intestinal flora; amino acids can serve as precursors for polyamine production (Seiler and Raul, 2007; Di Martino et al., 2013). Based on fecal sample analysis, naturally abundant polyamines include putrescine, spermidine, spermine and cadaverine in the human colon (Forget et al., 1997; Matsumoto and Benno, 2007). Putrescine, spermidine, and cadaverine are derived from the decarboxylation of ornithine, methionine, and lysine, respectively. Dysregulation of the level of polyamine and its amino acid precursors is connected with inflammation and autoimmune disorder (R. Wu et al., 2020). However, the underlying mechanism by which polyamine processed remains poorly uncovered.

Metagenomics has begun to study the composition and genetic potential of the gut microbiota in order to illustrate the breadth of the functional and metabolic potential of microbes. There is no doubt that there exists a link between constipation and microbiota (Ohkusa et al., 2019). However, there appears to be no available research that proves the involvement of the microbiota in relieving constipation following rhubarb extract treating. Accordingly, we performed metagenomics to demonstrate substantial metabolic differences across groups in order to assess the influence of constipation and rhubarb extract on the microbiota. On a side note, the microbiota would interact more directly with the host immune system and metabolism in the intestinal epithelium. Conceivably, the microbiota might theoretically be more directly engaged in causing constipation.

However, current research in relieving constipation by increasing bowel motility has been descriptive. Apart from that, the underlying mechanism by which rhubarb extract is possessed remains poorly addressed. Therefore, this study offers a substantial addition to studying constipation by revealing its fundamental process of understanding how the constitutive constipation response is regulated by rhubarb extract.

## 2 Materials

### 2.1 Animals

6–8-week old of C57B/6N male mice weighing 21 ± 1 g from the Laboratory Animal Services Center of Capital Medical University are raised under a standard environment (22.0°C–25.0°C, at a relative humidity of 50–70% under 12-/12-h light/dark cycle), and all procedures were carried out according to National Institutes of Health Guide for the Care and Use of Laboratory Animals (AEEI-2016–079).

### 2.2 Regents and dosage information

Compound diphenoxylate containing 25 mg of diphenoxylate and 2.5 mg of atropine sulfate monohydrate per tablet was purchased from Hefeng Medicine Industry (Guangxi, China, Lot: 210,704). The compound diphenoxylate was dissolved in normal saline to achieve an adequate concentration of 10 mg/ml. Administration of compound diphenoxylate to mice at the dose of 20 mg/kg *via* gavage lasting five days was prepared as a verifiable and repeatable constipation model group (Chen et al., 2019; Tan et al., 2021) as the constipation model group. We purchased rhubarb from Tongrentang Pharmacy (Beijing, China) and identified it with the assistance of Prof. W. Wang from Xuan Wu Hospital of Capital Medical University. As described previously (D. Wu et al., 2019), the roots of rhubarb were crushed and soaked in the annealing for 2 h, and diluted to 1 g/ml, and then stored at 4°C until use. Previous experiments studying strongly driven systems have reported remarkable effects at 600 mg/25 g. This is important because the optimal dose elicits alleviation without any other side reaction. Additionally, in an analysis of a large randomized clinical trial of constipation, the dose application was judged to be about 9-fold to those administered in human clinical trials adult dosage (Jiang et al., 2017).

### 2.3 Experimental design

**FIGURE 1:**
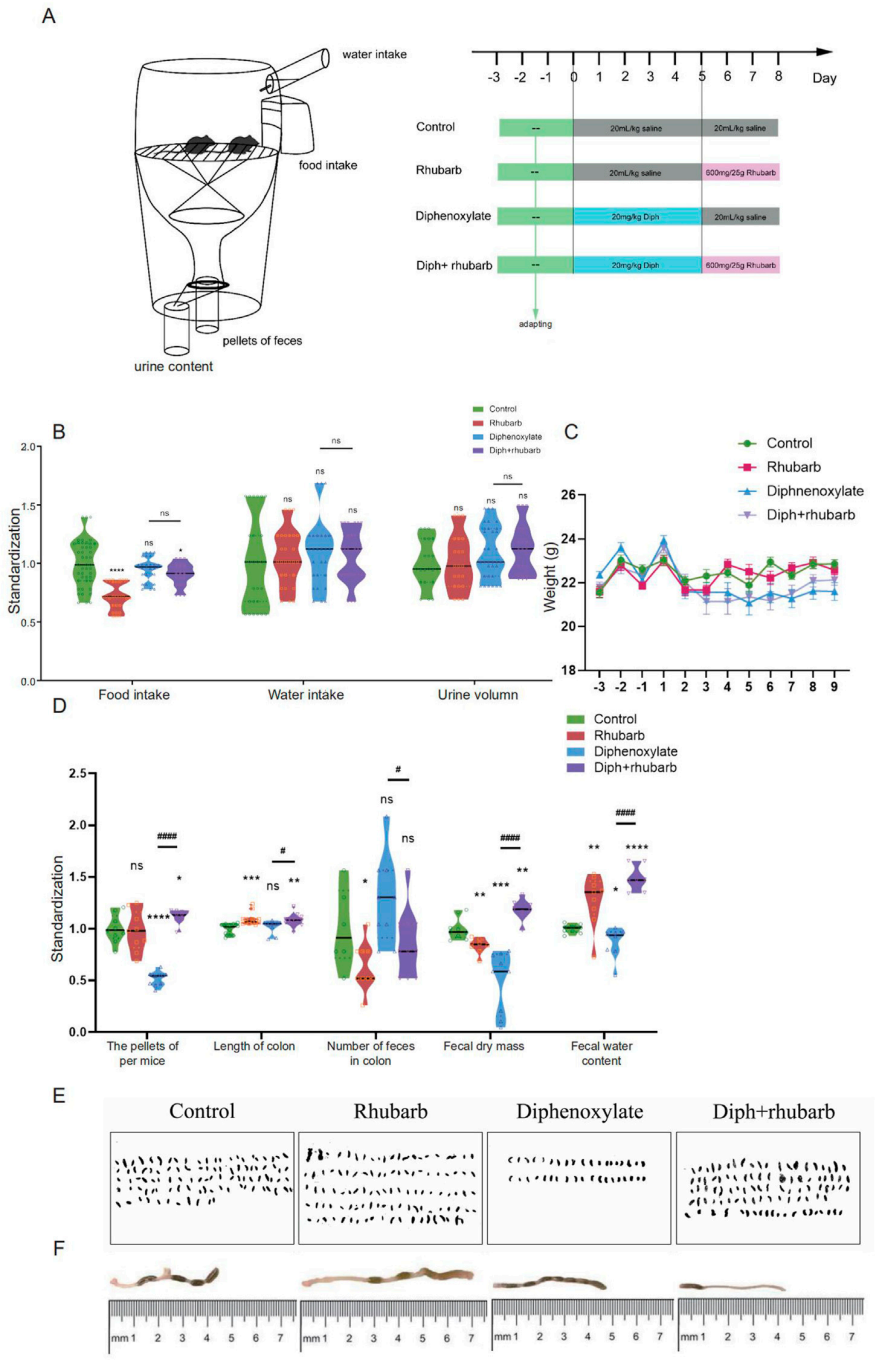
The experimental design and diphenoxylate-induced constipation paradigm with its reinstatement of rhubarb treatment. **(A)** A timeline detailing the pharmacological treatments and the experimental paradigm’s acclimation, grouping, and time points. **(B)** Assessments of the daily consumption of food and water intake, as well as urine volume. **(C)** Recording the body weight of mice fed with normal saline, rhubarb, diphenoxylate, and both diphenoxylate and rhubarb during the trial. **(D)** The violin graph represents the differences in the number of pellets defecated, fecal dry mass, fecal water content, and the length of the colon section, as well as the number of feces in the colon at the timeline of sacrifice in different groups. **(E)** Observation of the feces from one mouse per day to assess the feces characteristics in different groups. **(F)** Photographs of mouse colon and their lengths, as well as the amount of feces in them, are measured by the distance between the anus (at 0) and the cecum. All data from **(C–D)** is normalized by the control group. All values are presented as means ± SEM (*n* = 9 per group). ns, *p* > 0.05; *, *p* < 0.05; **, *p* < 0.01; ***, *p* < 0.001. * vs. control group; # vs. diphenoxylate group.

The mice were randomly divided into four groups with the equal number of animals in every group: the control group received normal saline alone on the same day as the other groups. Another group of mice was treated with normal saline vehicles once daily lasting five days, followed by three-day rhubarb extract followed at 600 mg/25 g. To induce constipation, mice undergoing administration of diphenoxylate for five consecutive days were separated into two groups, one with three-day normal saline treatment, and one co-administration rhubarb extract for three days. All mice were reared in the metabolic cage with free access to food and water to collect 24-h feces and supervised the consumption of food and water so as to accurately judge the success of model mice (Shan et al., 2010; Xu et al., 2012; Wang et al., 2014). Detailed information on experimental design is available in exhibited Figure 1A.

At the end of the experiment, feces from the colon, urine from the bladder, and blood were collected before euthanasia. At the end of the sacrifice, the colon was collected and dissected to be cut open for measuring colon length and other downstream analyses. Blood samples were collected and then centrifuged at 12,000 r/min for 30 min at 4°C to obtain the serum, which was subsequently stored at −80°C. The feces were stored in the sterile centrifuge tubes at −80°C until being performed 16 S rRNA and further metagenomic analysis. The colon tissue was removed from the point of 0.5 cm above the anus to the top of ileoceca and soaked in different fixative solutions. The tissue samples were fixed in 10% formalin overnight at room temperature and stained with Masson trichrome, Sirius Red, and hematoxylin and eosin (HE) for light microscopy interstitial image files. The samples were paraffin-embedded, and 5-µm-thick serially sections were mounted on glass slides and stored at −20°C. Before antigen retrieval, paraffin sections were first dewaxed, rehydrated through a graded alcohol series, and transparently with xylene.

## 3 Methods

### 3.1 HE staining

The sections were stained in parallel by modified Lillie-Mayer’s hematoxylin for 1 min, differentiated with 1% hydrochloric acid alcohol for 2–5 s, soaked in tap water for 10 min to turn blue, and followed by being dyed with water-soluble red dye for 1 min as indicated.

### 3.2 Masson trichrome staining

To evaluate whether collagen fibers are changed throughout this process, Masson’s trichrome staining was performed with a commercial kit (Beijing Solarbio Science and Technology Co., Ltd.). The sections were stained using Wiegert’s iron hematoxylin solution (Wiegert solution A: Wiegert solution B = 1:1) for 10 min and then stained with azulene for 10 min at room temperature, respectively followed by weak acid solution (deionized water: weak acid = 2:1) immersed in phosphomolybdic acid for 2 min, and stained in diluted toluidine blue for 1 min. All slices were rinsed five times with mild acid solution until the collagen fibers to the total area appeared blue. Similarly, the collagen fiber densities and distribution were quantified with Image-pro plus.

### 3.3 Sirius Red

Sirius red staining was detected for histologically assessing collagen level followed by a combination of microscopic including polarized light and optical microscopy. The sections were rehydrated and stained for 1 h with a Sirius red stain kit (0.1% Sirius red in a saturated aqueous solution of picric acid) (Beijing Leagene Biotechnology Co., Ltd.)

### 3.4 Atomic force microscopy

The muscle layer variation was observed in the response to different treatments, and we wonder whether the muscle change, along with the strength change, has a vital role to play. Therefore, AFM was implemented using a Multimode/Nanoscope IIIa AFM (Digital Instruments/Veeco, Santa Barbara, CA). MLCY-BIO (BRUKER, United States) with a nominal spring constant of 0.14 N/m, which is capable of detecting samples concerning their stiffness, adhesion, and modulus. The colon specimen was removed immediately, embedded in OCT, snapped frozen in liquid nitrogen, and substantially restored at −80°C. The colon tissue were sliced into 5 μm thickness. The pieces were fixed with 4% PFA for 30 min and rinsed with PBS mixed with 1% cocktail (27423400, Switzerland) for 5 min for a total of three times. These tissues went imaged under the AFM imaging system. All images were detected in the intermittent contact mode in regime liquid at room temperature.

### 3.5 Enzyme-linked immunosorbent assay

Levels of cytokines in the serum of all the mice (IL-15, IL-17A,IL-22, IL-23, and CCL5) were performed using commercially available mouse ELISA kits according to the protocols supplied by the manufacturer and detected by a multimode microplate reader (Beckmancoulter UniCel DxC 600 Synchron, United States).

### 3.6 *In vivo* paracellular permeability assay

To assess colonic paracellular permeability *in vivo*, mice were deprived of food for 18 h, then orally gavaged with 440 mg/kg body weight of FITC-labeled dextran (FD4) (Sigma, St. Louis, MO, United States). The mice were euthanized 4 h later, plasma was collected, its fluorescence intensity in serum was detected by a fluorescent microplate reader (excitation at 480 nm and emission at 520 nm; HTX Multi-Mode reader, SYNERG).

### 3.7 Real-time PCR analysis

Total RNA was extracted from prepared tissue using FastPure Cell/Tissue Total RNA Isolation Kit V2 (RC112, Vazyme, Nanjing, China) according to the product manual. The concentrations of isolated RNA were quantified by NanoDrop 2000 spectrophotometer (Thermo Fisher Scientific, Waltham, MA), and then reverse transcription was performed by HiScript III RT SuperMix for qPCR kit (R323, Vazyme, Nanjing, China) by BIO-RAD iCycler (BIO-RAD, United States). Finally, the cDNA was with Taq Pro universal SYBR qPCR Master Mix kit (Q712, Vazyme, Nanjing, China) by the CFX96TM Real-Time System (BIO-RAD, United States). The thermal cycles were 95°C for 5 min, 56°C for 15 min, 72°C for 10 min, for 45 cycles, and 60°C for 1 min. The relative amount of the target mRNA was normalized to the GAPDH level, and data were calculated by the 2^−ΔΔCT^ method. The primer sequences were listed as follows.

### 3.8 LC-MS/MS metabolite analysis

Targeted feces metabolomics quantifying fatty acids was performed by LC-MS/MS processes as previously reported by Gao et al. (2021). Fecal samples were quickly homogenized in a Bullet Blender into suspension. Hydrochloric acid (30 mM) was added, isotopically-labeled acetate (0.125 mM), butyrate hexanoate (0.125 mM), and 250 ml of Methyl tert-butyl ether (MTBE). Finally, each sample is a mixture of 400 ml in volume. Subsequently, the mixture was briefly mixed by vortexing for 10 s at 4°C twice, and the solvent layers were separated by centrifugation for 1 min 10 ml of MTBE operated from the samples was laterally transferred to an autosampler vial for GC-MS analysis by placing it in a separate auto-sampler vial to get a series of calibration standards for normal quality purposes. GC-MS analysis of samples was implemented with Agilent 69890N GC-5973 MS detector with the parameters given in extended methods. A 1 μL sample was injected with a 1:10 split ratio on a ZB-WAXplus, 30 m 0.25 mm × 0.25 μm (Phenomenex Cat# 7HG-G013-11) GC column. Helium was used as the carrier gas at a flow rate of 1.1 ml/min with 240°C as the injector temperature, and the column temperature was kept at 310°C under isocratic conditions. Quantification data were extracted and analyzed by MassHunter Quantitative analysis version B.07.00. SCFAs were normalized to the nearest-isotope labeled internal standard and quantitated using 2 replicated injections of 5 standards to create a linear calibration curve with accuracy greater than 80% for each standard (Roehsig et al., 2010).

### 3.9 DNA sequencing and metagenomic sequencing

Stool samples were collected in sterile tubes and immediately frozen before being kept at −80°C until performing further analysis. DNA was extracted with HiPure Stool DNA Kit (Shanghai Ponsure Biotech, China) and measured concentration and quality. Quantitative real-time PCR was performed using bacterial primers, which were targeted amplification of the combined V3 and V4 regions of the 16 S rRNA gene. Amplification was carried out using fusion primers containing the 16 S-only V3-V4 sequences fused to Illumina adapters overhung nucleotide sequences (Allen et al., 1984) and finally pooled and sequenced on Illumina’s MiSeq/NovaSeq platform at the Genomic and Proteomic Core Laboratory in Genewiz, LTD, Suzhou, China. The generated NGS data was filtered and clustered into operational taxonomic units (OTUs), which carry species distribution information. We applied weighted- and unweighted-UniFrac to compare the distances among the four groups. Principal coordinates analysis (PCoA) was used to screen out β diversity.

The metagenomic DNA in the colon content of mice in each group was extracted and quantified using the Stool Genomic DNA Kit (CoWin Biosciences, China). The purified DNA was then end-repaired using the End-it End-repair kit, and added an “A” base to the 3′end of DNA fragments. Furthermore, for adaptor ligation, paired-indexed Illumina dual-end adapters were replaced with palindromic forked adapters with unique 8-base index sequences embedded within the adapter and added to each end. Target DNA fragments within a specific length range were screened by the magnetic beads, and amplified with PCR with the index at the end of the target fragment to complete the construction and detection of the sequencing library. We prepared sequencing libraries using Illumina’s TruSeq ChIP Library Preparation kit, and barcoded libraries based on an Illumina HiSeq2000 instrument according to the fragment size. Lastly, we generated gene profiles using a gene catalog and estimated these data by the data library KEGG (Kyoto Encyclopedia of Genes and Genomes) ortholog (www.genome.jp/kegg/).

## 4 Results

### 4.1 Effects on the feeding behavior and stool parameters

To begin, the facts regarding the amount, weight, and water content of the fecal pellets are the most obvious indices to measure constipation under laboratory circumstances to determine whether diphenoxylate and rhubarb extract treatment changes eating behavior and excretion characteristics (Rasmussen and Pedersen, 2010). Therefore, alterations in food intake, water consumption, urine volume, and stool parameters were also measured daily in the four groups mentioned above. As shown in Figure 1B, food intake was dramatically reduced after treatment with rhubarb extract or diphenoxylate, respectively, in comparison to the control group, and the impact was returned to near-normal levels by treating constipation mice with rhubarb extract, but no significant difference in water intake and urine volume among the various groups. While a slight bodyweight decrease was observed in constipation induced by diphenoxylate, an enhancement after administering rhubarb extract was shown (Figure 1C). To address this issue, the fecal pellets of excreted daily collected from metabolic cages were evidently decreased in the model group compared to the control group (Figure 1D, *n* = 9, *p* < 0.001), while the pellets enhanced with rhubarb extract were administered. Furthermore, varied fecal color and shape are essential measurements of constipation. As shown in Figure 1E, the feces were irregular in size and shape with the variably gray color in the constipation group. At the same time, the pellets got washy or even unshaped after treatment with rhubarb extract. However, these classical studies confirmed a significant increase in the water content of feces in the rhubarb group (Figure 1D, *n* = 9, *p* < 0.01) compared to the pellets of feces (Figure 1D, *n* = 9, *p* = 0.7437). The feces in the constipation group had the most negligible water content and were remarkably enhanced after being treated with rhubarb extract. Also, we observed the number of feces in the colon was extremely reduced in the rhubarb group as compared to the control group, as shown in Figure 1D (*n* = 9, *p* < 0.01), which may contribute to explaining the more general phenomenon in the constipation mice treatment with rhubarb extract. Next, we determined whether the functional defecation was accompanied by abnormal alterations of intestinal length. As Figure 1F indicated, the measurement of colon length from ileocecum to distal colon in each mouse showed significantly longer colons in all rhubarb-treated mice, regardless of in normal mice or in constipation mice (Figure 1D, *n* = 9, *p* < 0.01), but no apparent differences was observed between constipation group and the control group. Overall, these results validated that the constipation models were successfully achieved, and rhubarb extract had evolved defecation benefits by partly enhancing the fecal water content. Changes in weight loss, fecal water content, and a number of defecation granules are also sensitive and responsible for the phenotype caused by rhubarb extract.

### 4.2 Alterations of histopathological and cytological structure of colon

**FIGURE 2:**
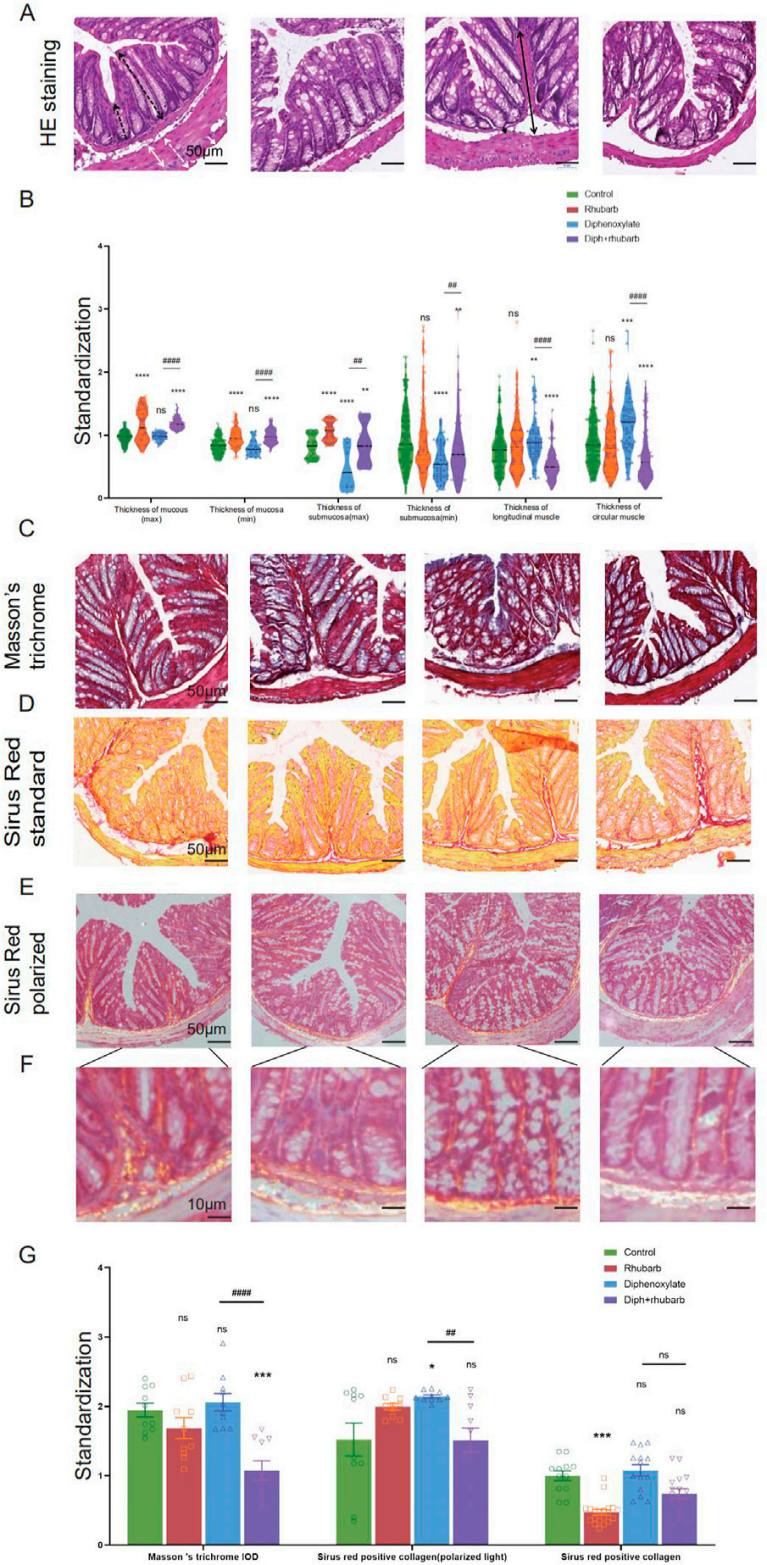
Histochemical colonic tissue stains reveal some routine qualitative differences for comparative purposes across the four groups. **(A)** H&E staining reveals the general characteristics presented in a comprehensive overview. The black dotted line with double arrows: mucus layer (max). Black dotted line with single arrows: mucus layer (min). The black line with double arrows: submucosa layer (max). Black line with single arrows: submucosa layer (min). A white dotted line with double arrows: muscle layer. A white line with double arrows: circular muscle layer. White line with single arrows: longitudinal muscle layer. **(B)** The violin graph displayed the quantitative analysis of the thickness of the mucus layer (max and min), submucosa (max and min), and muscle layer (circular and longitudinal muscle). The data were normalized by the control group. **(C)** Representative images of Masson’s Trichrome staining to assess the fibrotic changes in the colon. **(D)** Total collagen in colon sections is stained with Sirus Red. Representative images are shown with a magnification of × 10. Representative picture of Sirus Red staining in polarized light, magnification, × 10. **(E–F)** Sirus Red-stained sections observed with polarized light, magnification × 20. Scale bars, 50 μm. **(G)** The bar graph is quantified analysis of interstitial fibrosis and the total collagen amount by Sirus Red staining. The figures are successively from the control group, rhubarb group, diphenoxylate group, and Diph + rhubarb group. All data are presented as means ± SEM (n = 9 mice per group). ns, *p* > 0.05; *, *p* < 0.05; **, *p* < 0.01; ***, *p* < 0.001. * vs. control group; # vs. diphenoxylate group.

We investigated the associated changes in the histopathological and cytological structure of the colon induced by constipation and rhubarb extract challenge. We examined intestinal epithelial information employing H&E staining (Figure 2A). First of all, alterations in thicknesses of the colonic mucosa, submucosal, and muscle layer were analyzed (Qu et al., 2017). The results showed that the layered muscle structure of the mouse colon under constipation status became thicker (Figure 2B, *n* = 9, *p* < 0.001), which is markedly reduced after the treatment constipation model with rhubarb extract (Figure 2B, *n* = 9, *p* < 0.001). A contrary trend was detected in the thickness of the mucosa layer and rhubarb extract treatment induced the enhancement of the mucosa layer (Figure 2B, *n* = 9, *p* < 0.001). The thickness of the mucosa layer in constipation, whereas did not differ compared to the control group (Figure 2B, *n* = 9, *p* > 0.05). There was a significant decrease in the thickness of the submucosal layer in the constipation group compared to the control group, and this trend was reversed by the treatment of rhubarb extract (Figure 2B, *n* = 9, *p* < 0.001).

Considering that the muscle layer had a noticeable impact on the contraction, we conjectured that fibrosis might be an advanced-stage phenotype regulated by collagen fiber rather than an early causal factor in developing hardness increases. To uncover whether fibrosis was involved in the process, all of these samples were observed by Masson’s trichrome and Sirius red staining. From Figure 2C, using Masson’s trichrome staining, we can see the constipation group contained more fiber, while it had less after rhubarb extract administration compared to the control group. In line with the results, what is interesting about the data in Figure 2D using Sirius Red staining was that quantification of collagen deposition showed a remarkably reduced following treatment with rhubarb extract. However, the polarized result revealed that the fiber was markedly increased, while the rhubarb group did not exhibit decreased tendency. To conclude, these data provided strong evidence that collagen fiber over-expression plays a causal role in increasing contraction intensity in muscle, which in turn, leads to a decrease in muscle strength.

**FIGURE 3:**
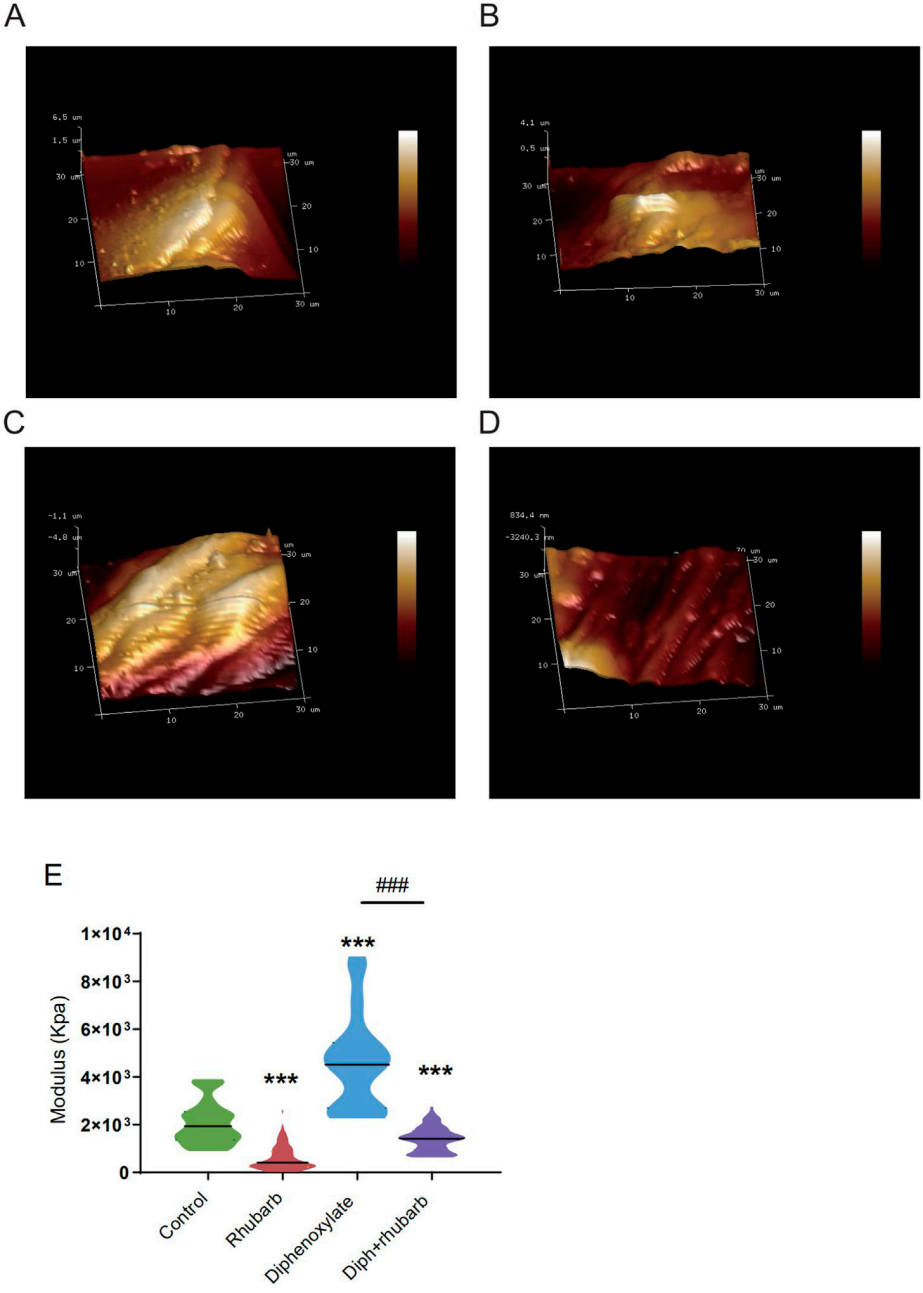
Atomic force microscopy (AFM) 3D images for the four groups: control group **(A)**, rhubarb group **(B)**, diphenoxylate group **(C)**, and Diph + rhubarb group **(D)**. **(E)** The violin graph indicates the modulus of the colon muscular from four groups. ns, *p* > 0.05; *, *p* < 0.05; **, *p* < 0.01; ***, *p* < 0.001. * vs. control group; # vs. diphenoxylate group.

To further validate these dominant effects on fiber, we assessed the strength and modulus of collagen fiber utilizing AFM. The elastic moduli of smooth muscle, especially in the digestive tract, are still largely unexplored. An accurate mean modulus can be obtained only the thickness of the colon tissue section is known. This value is based on the mean tissue rupture force and deformation of intestinal smooth muscle under fresh frozen sections. MLCT-BIO was chosen for the characterization. Figure 3A–D showed the average elastic moduli of control, rhubarb, diphenoxylate-induced constipation, and constipation model treatment with rhubarb extract measured by using MLCT-BIO. As shown in Figures 3B, E, the group in rhubarb had the lowest modulus of 580.9 ± 111.4 KPa, while the group in constipation showed a much higher modulus of 4,663 ± 305.2 KPa (Figure 3C, E). Notably, as Figures 3D, E suggested, the moduli in the group of constipation treatment with rhubarb extract showed a sharp decrease to 1,396 ± 219.6 KPa (Figure 3D, E). All these data were compared to the modulus in the control group. It was noted that all results of the modulus in constipation showed a significant increase, indicating that the elasticity of smooth muscle would also be significantly increased. This change, due to a physiological point of view, was consistent with the previous increase in collagen fibers to eliminate stool in the colon. Simultaneously, it was also observed that after the use of rhubarb extract, the content of collagen fiber in the intestinal tissue was quickly decreased due to the increase of moisture in the intestinal tract and the increase in movement speed, indicating that its elasticity would also be correspondingly increased. This change was consistent with the decrease in the content of collagen fiber measured in the previous experiment. Therefore, slight elastic changes in smooth muscle tissue caused by changes in collagen fibers can be assessed by atomic mechanics microscopy modulus.

### 4.3 Measurement of cytokine concentrations

**FIGURE 4:**
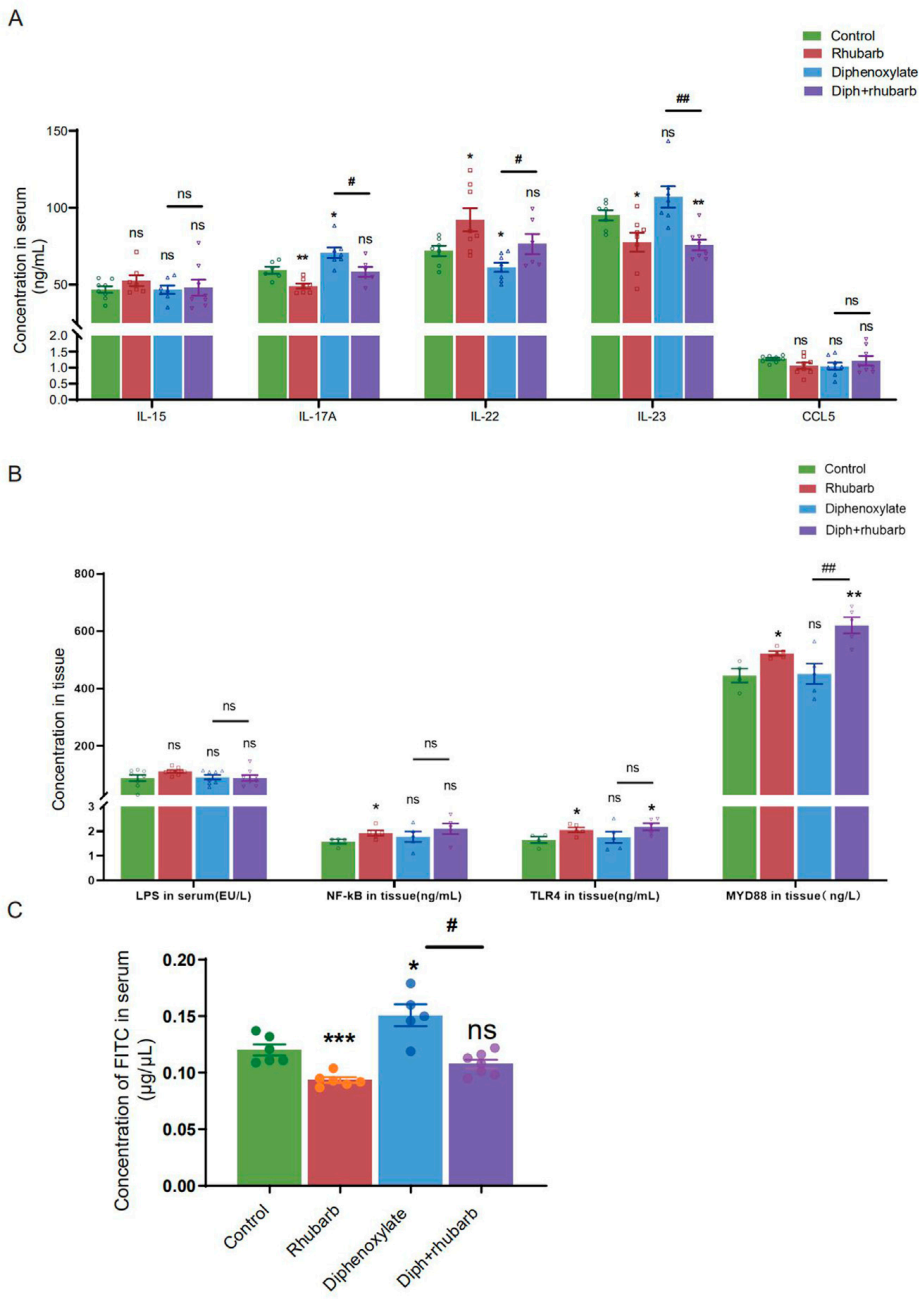
Measurement of the cytokine levels and paracellular barrier function by paracellular tracer flux assays using FITC-dextran (40,000) among the four groups. **(A)** The bar graph depicts the cytokine concentrations, including IL-15, IL-17A, IL-22, IL-23, and CCL5 in the plasma. There is no obvious change as visualized distinct patterns in the IL-15 as well as CCL5 among the four groups. **(B)** The bar graph exhibits the concentrations of LPS, NF-κB, TLR4, and MyD88 in colonic tissue by ELISA among the four groups. **(C)** The concentrations of FITC-dextran in the circulating plasma are measured in four groups. The figure represents the combined results of repeated twice with similar results, which are expressed as the means ± SEM of six to eight mice per group. ns, *p* > 0.05; *, *p* < 0.05; **, *p* < 0.01; ***, *p* < 0.001. * vs. control group; # vs. diphenoxylate group.

Colon crypt mucin has been found to be regulated by cytokines (Iwashita et al., 2003; Schwerbrock et al., 2004). The change in the submucosa layer indicated that inflammation might be involved. To further elucidate the complex relationship between constipation and inflammation in intestinal epithelial cells, we sought to determine the expressions of some cytokines such as IL-15, IL-17A, IL-22, and IL-23. IL-15, with pro-inflammatory effects, however, there was no difference among the four groups, as Figure 4A indicated. IL-17 recruits neutrophils into the cecal mucosa to protect from the invasion of bacteria, but induces excessive inflammation (Huang, 2021). In alignment with our expectation, constipated mice predisposed to induce inflammation cause IL-17A to be the highest. In addition, the high concentration decreased after treating constipated mice with rhubarb extract. Amongst the current research, the prevailing view is that IL-22 is mainly related to the maintenance of mucus barrier function by promoting Lgr5^+^ epithelial stem cell regeneration/proliferation (Lindemans et al., 2015). And the results indicated that the IL-22 concentration in serum dropped in the constipation group and peaked in the rhubarb group, which implied that rhubarb might play a protective role through increasing IL-22 levels. IL-23 induces neutrophil polarization and promotes inflammation. As Figure 4A shows, we identified a noticeable decrease of IL-23 expression in the groups administered rhubarb extract regardless of the control mice or the constipation mice. In parallel to the above pro-inflammation cytokine, the constipation group had the highest level of IL-23.

To further determine the role of constipation on inflammation, we widened our search to screen for the concentration of lipopolysaccharide (LPS) in serum and its receptors, Toll-like receptor 4 (TLR4) and myeloid differentiation primary response gene 88 (MyD88), in tissue (Figure 4B). The results of nuclear factor-κB (NF-κB), TLR4, and MyD88 were coincident. More precisely, the level in the treatment with the rhubarb group was particularly increased compared to the control group and dropped in the constipation group.

### 4.4 Impaired epithelial barrier function in constipation mice

The mucus layer serves as a vital physical barrier against both microbiota and toxins. Damage to gut barrier integrity, including the mucus layer, epithelial cell junctions, and AMP secretion, for example, has been proven to link to IBD pathogenesis. As the readout of intestinal barrier function (Figure 4C), we detected the intestinal permeability evaluated by serum FITC-dextran concentration 4 h after oral gavage was significantly enhanced in constipation mice than to controls (*n* = 4-6, *p* < 0.05). Notably, rhubarb extract treatment had decreased considerably intestinal permeability (*n* = 4-6, *p* < 0.05). The above results indicated that the intestine barrier was more prone to vulnerability in the constipation group, and more integrity following rhubarb extract administered.

### 4.5 Metagenomics analysis

**FIGURE 5:**
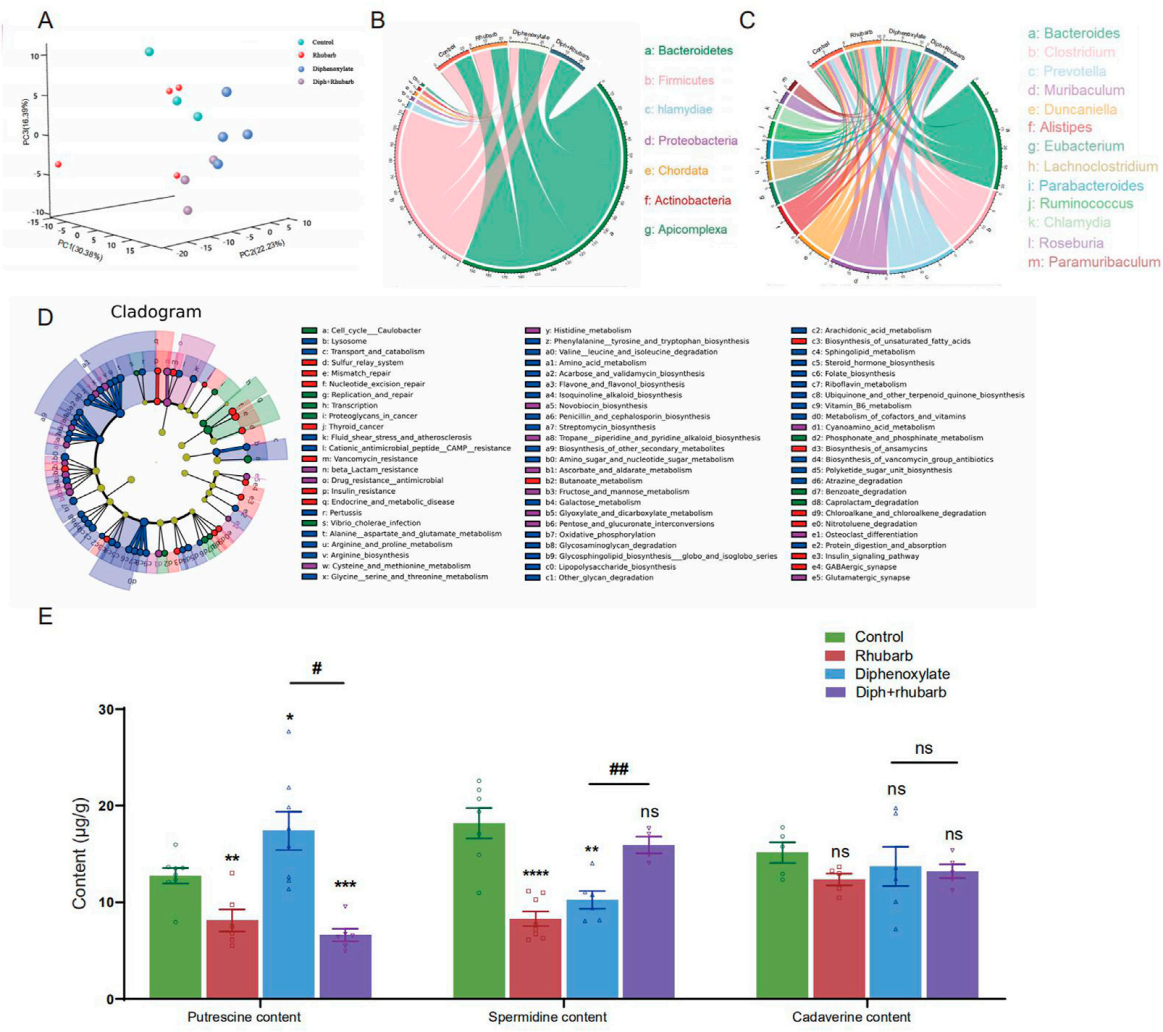
Metagenomics analysis of the feces collected from four groups’ fresh colon and measurements of biogenic amine. **(A)** PCA of 16 S metagenomics data from the microbial population in the feces of the four separate groups with three to four animals each. Comparison of community diversity based on different metric distances beneficial for microbial communities encoding taxonomic profiles into kernel matrices. **(B)** *Circus* data reveals the microbiome composition at the phyla level in distinct groups. (*n* = 3 or 4). **(C)** *Circus* data shows the microbiota composition in distinct groups at the genus level. (*n* = 3 or 4). **(D)** Functional characterization of microbiota based on metabolic pathway abundances. A cladogram represents the KEGG BRITE functional hierarchy, with the outermost circles representing individual metabolic modules and the innermost tiny circles indicating the KEGG BRITE functional hierarchy. **(E)** The bar graph illustrates that putrescine, spermidine, and cadaverine are measured in the feces (*n* = 6 or 8). The data is shown as the mean ± SEM. ns, *p* > 0.05; *, *p* < 0.05; **, *p* < 0.01; ***, *p* < 0.001. * vs. control group; # vs. diphenoxylate group.

To further reveal the functions and metabolic pathways regulated by constipation and rhubarb extract treatment, we performed metagenomics analysis. We detected the top 30 bacterial phyla, classes, orders, families, genus, and species in each sample. As the PCoA dipicted in Figure 5A, the distribution of the four groups was significantly different. Constipation induces a considerable change in the diversity and abundance of the gut microbiota composition at the species level, as evidenced by decreased relative abundances of Firmicutes and increased relative abundances of Bacteroidetes in feces (Figure 5B). The ratio between these two phyla (the Firmicutes/Bacteroidetes (F/B) ratio) has been linked to homeostasis, and a drop in this ratio can lead to gut inflammation (Stojanov et al., 2020). In the constipation group, the percentage of Firmicutes decreased from 26.69% to 16.61%, whereas the percentage of Bacteroidetes increased substantially from 24.4% to 32.76% *versus* the control group. Surprisingly, the changes were partly diminished by treating constipation mice with rhubarb extract. The addition of rhubarb extract in the normal group, in the other hand, imposed little impact on the abundance of *Bacteroides* and Firmicutes. Similarly, the F/B remarkably decreased after exposure to constipation and increased with the addition of rhubarb extract. These findings may imply that constipation was more prone to inflammation, and the tendency was probably reversed by rhubarb extract treatment.

Furthermore, we cataloged the genes in the genus level to reveal discrepancies (Figure 5C). Alistipes and *Trichinella* levels reduced in normal mice after three days of rhubarb extract administration, but Duncaniella, lachnoclostridium, and Parabacteroids level increased. *Bacteroides* and Muribaculum were enhanced in constipated mice, but *Clostridium*, Roseburia, and Ruminococcus were markedly reduced. When the constipation mice were given rhubarb extract, the level of Alistipes, Muribaculum, and Prevotella decreased while *Clostridium* and Lachnoclostridium increased. This evidence corroborated that the Diph + rhubarb group was not completely equivalent to the control group. Alistipes and Ruminococcus were at evidently higher abundant than the control group, although *Bacteroides*, Muribaculum, and Parabacteroids were at lower levels.

In addition to relative abundances of microbiota, we detected the abundances of microbial metabolic pathways as profiled *via* metagenomic shotgun sequencing of a subset of the available body habitats. To identify biological pathways that are regulated by the diversity of the microbiomes, we annotated the genes based on KEGG databases. As for the KEGG databases (Figure 5D), the gene catalog mainly assigned the top KEGG categories: metabolism, genetic information processing, environmental information processing, cellular process, human diseases, organismal systems, and drug development. The results revealed that KOs in the rhubarb group were more abundant in those involved in glycolysis/gluconeogenesis (KO00010) and oxidative phosphorylation (K00190) and less abundant in those involved in quorum sensing (KO02024), DNA replication (KO03030) and homologous recombination (KO03440) compare to that in the control group. When constipation, the KOs participate in galactose metabolism (KO00052), oxidative phosphorylation (KO00190), and glycine serine and threonine metabolism (KO00260) were up-regulated, while the KOs involved in quorum sensing (KO02024) and mismatch repair (KO03430) were down-regulated. In the Diph + rhubarb group, the KOs representing glycolysis/gluconeogenesis (KO00010), purine metabolism (KO00230), and Aminoacyl-tRNA biosynthesis (KO00970) were less abundant, while the KOs taking part in the quorum sensing (KO02024) and RNA degradation (KO03018) were more abundant compared to the constipation group. However, the up-regulation of functions involved in oxidative phosphorylation, alanine aspartate and glutamate metabolism, and biosynthesis of amino acid was remarkable and the down-regulation of functions partaking in DNA replication and mismatch repair was significant. These gene expression changes are statistically significant, with false discovery rates below 0.01.

### 4.6 Treatment of constipation with rhubarb caused changes in biogenic amines

Based on the KEGG analysis, amino acid metabolism plays an essential role in constipation. Moreover, our current findings also suggested that constipation had a major impact on the gut microbiome and fatty acids (Gao et al., 2021). It is worth mentioning that biogenic amine is closely associated with microbiomes. Upon the fecal sample analysis, putrescine, spermine, spermidine, and cadaverine are the most common in the human colon (Kim et al., 2013; Holmes et al., 2017). Therefore, we investigated putrescine, spermidine, and cadaverine through bioinformatics analysis, as shown in Figure 5E. It was reported that *in vivo* and *in vitro*, putrescine has been shown to impair intestinal barrier function by disrupting tight junction integrity, aggravates gut leakiness, and subsequently causes disease susceptibility during colonic autoinflammation and infection (Grosheva et al., 2020). In line with our expectations, the amount of putrescine increased in the constipation group and dropped to a lesser extent after exposure to rhubarb extract. Using transcriptome and microbiome analysis, spermidine was found to play a role in maintaining a protective gut microbiota by reducing the expression of genes encoding for a-defensins (DEFAs) (Gobert et al., 2022). In contrast to putrescine, spermidine content in the constipation was lower than in the control group. Likewise, rhubarb extract contributed to enhancing the abundance of spermidine, causing the level to peak in the group treated with rhubarb extract alone, and administration of rhubarb extract may enable a considerable reversal of this decreasing effect of constipation on spermidine. Cadaverine, one of a family of small aliphatic nitrogenous bases (polyamines), may be proposed to have the potential to promote bacterial survival under antibiotic exposure and tolerance/resistance formation (Samartzidou and Delcour, 1999). However, none of these differences were statistically significant among the groups.

### 4.7 SCFA and MLCFA

**FIGURE 6:**
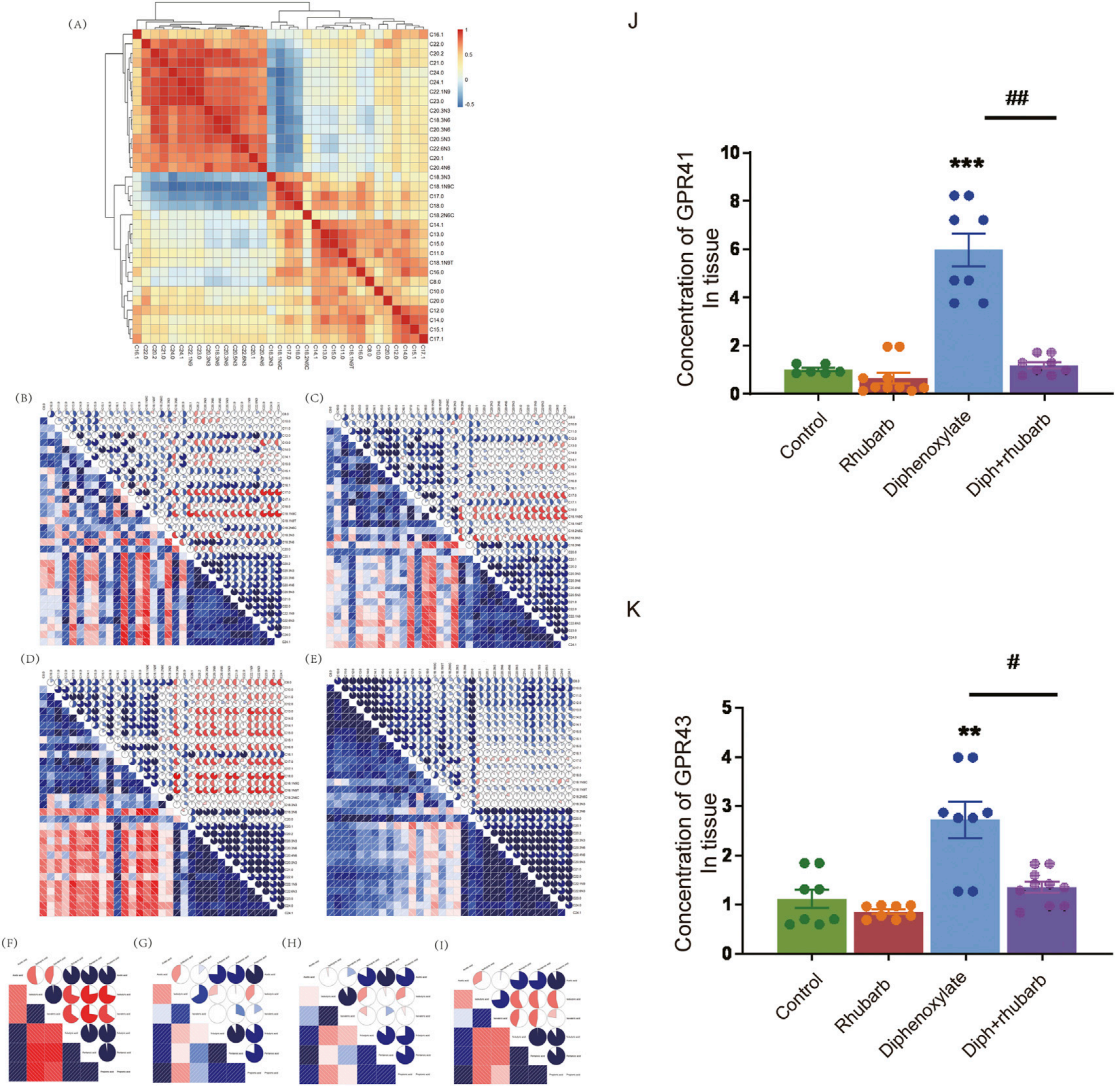
Effect of rhubarb and compound diphenoxylate on the changes of MLCFAs and SCFAs in the feces. **(A)** Spearman rank correlation matrix of the C10-C24 medium-long chain fatty across all samples. The colors denote the correlation coefficients, with one indicating a perfect positive correlation (red), and -1 indicating a perfect negative correlation (blue). This clustered heatmap was created using the R package “pheatmap.” version 1.0.12. https://CRAN.R-project.org/package=pheatmap. **(B–E)** Correlograms of the C10-C24 medium-long chain fatty in the normal group **(B)**, rhubarb group **(C)**, diphenoxylate group **(D)**, and Diph + rhubarb group **(E–I)** Correlograms of the matrix of the short-chain fatty in the normal **(F)**, rhubarb **(G)**, constipation **(H)** and Diph + rhubarb group **(I–K)** The bar graph illustrates the concentrations of SCFA receptors, GPR41/GPR43, from the four groups. The data is shown as the mean ± SEM. ns, *p* > 0.05; *, *p* < 0.05; **, *p* < 0.01; ***, *p* < 0.001. * vs. control group; # vs. diphenoxylate group.

Microbes must be metabolically active to survive in the gut environment rather than merely remaining inside it. Research has pointed out that the intestinal flora has an effect on the progress of composition and amount of various microbes, food debris as well as fermentation products such as MLCFAs or SCFAs (Khalif et al., 2005). Gut microbes play an integral role in physiological regulation, facilitating metabolism, influencing immunity, and remaining gut function (Khalif et al., 2005). Changes between SCFAs and constipation have frequently been reported (Gao et al., 2021), although there is no link between MLCFA and SCFA with rhubarb extract treatment in constipation models. Notably, fatty acid metabolism was involved in the KEGG analysis. To further reveal the association between fatty acids and constipation, we analyzed clustered heatmap based on the Spearman rank correlation matrix. In contrast, a hierarchical cluster analysis of all the samples was performed on the correlation coefficients between each pair of fatty acids across all samples (Figure 6A). The fatty acids, which had the analogous correlations with other fatty acids, were placed close in location. Notably, it can be seen that the four fatty acids (C18.3N3, C18.1N9C, C18.0, C17.0) were clustered into one group, all of which were negatively correlated to another fatty acids group (including C16.1, C18.3N6, C20.1, C20.2, C20.3N3, C20.3N6, C20.4N6, C20.5N3, C21.0, C22.0, C22.1N9, C22.6N3, C23.0, C24.0, C24.1). Following that, we performed the MLCFA correlograms for four groups. There were remarkable disparities between the four groups. Constipation caused a significant modification of the interconnections between MLCFA, and the median correlation coefficients (0.2097902) (Figure 6B) was substantially different from the normal group (0.3356643, *p* < 0.001) (Figure 6D). In rhubarb group, there were 150 positive correlations dropped, 157 positive correlations increased, 59 negative correlations decreased, 50 negative correlations rose and 89 correlations altered in comparison of the normal group. Compared to the constipation group, 92 positive correlations decreased, 163 positive correlations increased, 48 negative correlations decreased, 67 negative correlations increased and 141 correlations altered in constipation. However, in the constipation case, 83 positive correlations declined, 165 positive correlations grew, 44 negative correlations reduced, nine negative correlations increased, and 178 correlations altered. There were 269 (46.30%) statistically significant correlations in the constipation group (Figure 6D). In contrast, there were 206 (35.46%) such correlations (*p* < 0.001) in the normal group (Figure 6B) and 232 (39.93%) in Diph + rhubarb group (Figure 6E), revealing that the MLCFAs in case of constipation were more interactive and recovered when pretreatment rhubarb extract. Furthermore, no remarkable alterations occurred when treated the rhubarb extract alone (199, 34.25%). Additionally, substantial relationships correlations (|r|>0.75) were found in 103 (17.73%) of the normal group, 115 (19.79%) in the rhubarb group (ns), 158 (27.19%) in the constipation group (*p* < 0.001) and 184 (31.67%) in Diph + rhubarb group (*p* < 0.001). These findings suggested that only little variations existed among the fatty acids that were longer than C20.0 (including C20.1, C20.2, C20.3N3, C20.3N6, C20.4N6, C20.5N3, C21.0, C22.0, C22.1N9, C22.6N3, C23.0, C24.0, C24.1) as shown in Figures 6B–E. The correlations of rhubarb group (Figure 6C) among the fatty acids longer than C8.0 and shorter than C20.1 altered considerably, compared to the normal group (Figure 6B). In line with the modifications, the varieties in correlation in the Diph + rhubarb group (Figure 6E) are also located in the same sites as those in the constipation group (Figure 6D), indicating that the changes of correlation among the C8.0 to C20.0 were remarkable. Strikingly, majority of the negative correlations in the constipation group had turned into positive correlations in the Diph + rhubarb group.

Finally, we revealed the SCFA correlograms in four groups. As shown in Figures 6F–I, there are 19 (90.48%) statistically significant correlations between SCFAs existed in the normal group and 13 (61.90%) in the other three groups. As indicated in Figure 6G, the correlations of isovaleric acid in the rhubarb group has a noticeable difference compared to those in the normal group (Figure 6F). The same conclusion was reached when the Diph + rhubarb group was shown in figure. 8I was compared to the constipation group (Figure 6H). The results above demonstrated that constipation and rhubarb had an effect on fatty acids.

### 4.8 Changes in the expression of GPR41 and GPR43 in different groups

**FIGURE 7:**
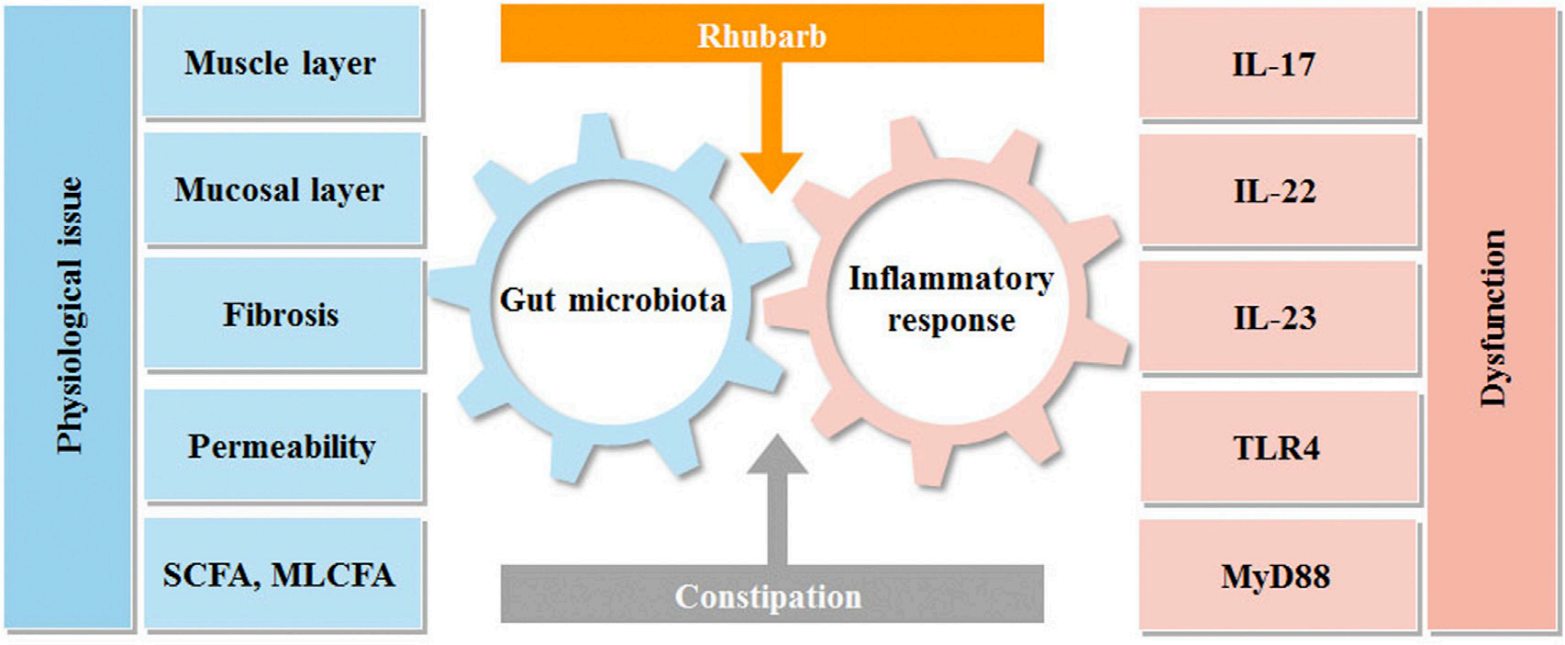
Interactions between rhubarb, constipation, inflammatory response and gut microbiota. Intestinal flora and immunity are in a constant state of flux. Constipation can upset this equilibrium, producing intestinal discomfort, which can lead to intestinal inflammation and bacterial imbalance. Rhubarb can help with constipation by modulating inflammatory reactions in the gut and the microbiota.

SCFAs may signal through cell surfaces, like GPR41, GPR43, and GPR109A, to activate signaling cascades and play a pivotal role in maintenance of intestinal inflammation. So we evaluated the expression of GPR41 and GPR43 in the colon tissue (Figures 6J,K). As Figure 7 shown, their expressions both increased dramatically in the constipation group. In contrast, no differences were demonstrated in the rhubarb group or the Diph + rhubarb group compared to the control group. It is noteworthy that their elevated expressions in the constipation group were rectified after being treated with rhubarb extract, which elucidated that rhubarb may have an anti-inflammatory impact.

## 5 Discussion

Rhubarb is an effective Chinese herb used to relieve constipation that has aroused much attention due to its great usage. To unveil the mechanism of relieving constipation by rhubarb extract, we generated the constipation model. In this study, our data suggested that the muscle layer and the new collagen in constipation were increased significantly, indicating that constipation may promote fibrosis. Of note, rhubarb has the capacity to reverse stiffness and restore muscular strength, as seen by a thinner muscle layer and less collagen. Moreover, our previous studies have revealed that promoting colonic mucus synthesis and secretion. Colonic mucus secreted from goblet cells is attached to the epithelium and isolates it from the external environment (Pelaseyed et al., 2014). The results above verify that rhubarb may relieve constipation by strengthening muscles and boosting mucin secretion to promote defecation.

As it depicted the interaction between intestinal flora and inflammation in Figure 7. Our data revealed that the constipation mice with the decreased mucus layer were prone to have impaired barrier and increased permeability with the manifestation of a high concentration of FITC-dextran. The condition also created an opportunity for germs to penetrate and induce inflammation, which accounted for the increased pro-inflammatory cytokines, such as IL-17A and IL-23. It is worth noting that the tendency of IL-22 was diverted as it peaked in the rhubarb group and fell in the constipation group. As the previous experiments demonstrated that IL-22 could contribute to retaining the mucus barrier function (Lindemans et al., 2015), the current findings supported our hypothesis that rhubarb contributed to the maintaining of intestinal barrier tightness and integrity.

MyD88, a fundamental role in the innate immune system, is the primary adaptor protein not only of IL-1 and IL-18 receptors but also of almost all the TLRs and thus considered a central hub of the inflammatory signaling cascades as well as is found to be required in LPS signaling. An interesting report concluded that IL22 induced significant upregulation of transcripts involved in microbial sensing (Tlr4, Myd88, Tnfaip3) (Powell et al., 2020). NF-κB, activated by TLR stimulation, is a crucial regulator of inflammation, innate immunity, and tissue integrity (Banoth et al., 2015; O'Neill et al., 2013). The tendency of LPS, NF-κB, TLR4, and MyD88 coincided in the four groups. The high expressions after being treated with rhubarb were uncommon due to their pro-inflammatory roles. However, several reasons may make sense. Firstly, rhubarb treatment arouses the activation of the mast cells, which plays an important role in immunity, as our previous report examined. On the other hand, the plasma cells were accumulated and activated, which is a critical step for innate immunity. Moreover, TLR/NOD ligands have been shown to modulate mucin gene expression and promote mucin secretion from goblet cells (Birchenough et al., 2016), which may be one of the mechanisms that rhubarb promote mucin secretion.

What is the role of gut flora in this process? Intestinal flora contains about 1,000 different bacteria (Turnbaugh and Gordon, 2009) and has a sophisticated effect on immunity and metabolism (Thaiss et al., 2016). It was emphasized that lots of diseases have demonstrated bacterial diversity along with reductions in the abundance of beneficial microorganisms due to the fragility of the gut microbiota. Many disorders have shown decreased bacterial diversity as well as decreases in the quantity of helpful microorganisms due to the fragility of the gut microbiota (Frank et al., 2007). Imbalances in the gut microbiota cause a chronic inflammatory response and increased susceptibility to viral and bacterial infections (Konturek et al., 2015). According to the PCA result, the number of gut microbial species, bacterial abundance, and flora diversity were all remarkably different in the four mice models. Specifically, the exposure to constipation enabled the potential of decreasing the diversity of the microbiome and characterized the high concentration of Bacteroidetes, and markedly reduced the F/B ratio. A stream of a pilot study reported the predisposition to inflammation sensitivity caused by the decreased F/B ratio. Microbes are metabolically active to survive in that gut environment rather than simply remaining within the gut. Hence, the gut flora would have an important influence in many areas, such as the composition and number of diverse bacteria, food debris, and fermentation products. To gain functional insights into colon metabolism, we assessed the genes by KEGG analysis. According to the variance analysis, it captured the preference for amino acid metabolism. In addition, glycometabolism was also involved in the process. Therefore, we applied the MS analysis to determine the alteration of biogenic amine and fatty acid. Polyamines have attracted much interest, in part, because of their essential roles in multiple cellular functions, like cell growth, mitochondrial metabolism, and histone regulation (Barker et al., 1987; Seiler and Raul, 2007; Pegg, 2009, 2016; Schiumerini et al., 2018; Oliphant and Allen-Vercoe, 2019; Tofalo et al., 2019). Gut microbiota can produce bacterial biogenic amines, including putrescine, cadaverine, tyramine, and 5-aminovalerate from amino acid degradation (arginine, lysine, tyrosine, and proline, respectively). It is beyond all doubt that our data elucidate that putrescine and spermidine significantly changed. It was later found that MLCFA and SCFA were altered. SCFA has an impact on maintaining homeostasis in the colon and supplies 60%–70% of the energy that colonic epithelia need (Khalif et al., 2005). Bacteria have the ability to create SCFAs. Notably, the amount of bacteria, pH, and substrate may all have a significant impact on the process (Zhu et al., 2014). Previous studies have shown that different substrates produce different amounts and proportions of SCFAs, which participate in many critical physiological, metabolic processes *in vivo*, such as induction of cell differentiation, regulation of the growth and proliferation of normal colonic mucosa, and reduction of the growth rate of colorectal cancer cells. As our investigation shows, the composition and diversity of the intestinal flora have varied. It can be seen that SCFAs, such as butyrate, including N-butyrate and isobutyrate, pentanoic acid, and isovaleric acid, increased significantly after rhubarb administration, but decreased considerably in the constipation group in our research. N-butyrate and pentanoic acid had a more significant decrease further in the treatment group, while isovaleric acid increased significantly. On the contrary, much of the research on this topic demonstrated that the content of isobutyrate in samples from subjects with constipation is significantly higher than in those from healthy people (Kang et al., 2015). The diet might make sense of the phenomena, given that SCFAs originate from the degradation of polysaccharides. Emerging evidence has come to suggest that SCFAs and MCFAs were mainly esterified by long-chain fatty acid groups, and SCFA and MCFA concentrations in full-term milk were significantly higher than those in premature milk (Dai et al., 2020). The correlation between SCFAs and MLCFAs in feces, especially in the alteration of intestinal flora, needs further study. Numerous studies have highlighted the importance of SCFAs in activating GPR41 and GPR43 on intestinal epithelial cells, resulting in mitogen-activated protein kinase signaling and rapid production of chemokines and cytokines (Kim et al., 2013). These pathways regulate protective immunity and tissue inflammation in mice. High level of GPR41 and GPR43 in constipation mice are in perfect agreement with the results regarding inflammation in constipation.

## 6 Conclusion

The most notable conclusion from this study is that constipation was linked to inflammatory response and gut microbiota as well as metabolic disorders. Notably, rhubarb treatment may play the regulatory and reversing role in these biological processes to alleviate constipation *via* a multitude of approaches. Undeniably, the major limitation of this study is that rhubarb was used in this experiment rather than its active component, which resulted in a convoluted impact. Notwithstanding these limitations, the study argues that this novel work should assist in our understanding the function of rhubarb as a potential multi-target drug for clinical application.

## 7 Statistical analysis

All data other than the sequencing data were plotted and analyzed with Prism Software 8.0 (GraphPad Software, San Diego) and presented as mean ± standard error of mean (SEM). Comparisons between groups were performed using ANOVA with *post hoc* tests or Student’s t-tests. A *p*-value less than 0.05 was considered statistically significant, and one between 0.05–0.10as showed a trend toward statistical significance. Principal component analysis (PCA) based on the unweighted UniFrac distance metrics was used to assess Beta diversity. Pearson r coefficients were applied to calculate bivariate correlations, and paired Mann-Whitney’ test was used to compare *p*-values between groups. Correlation matrices also were displayed as schematic correlograms (Daraio et al., 2003). All statistical analyses were performed in Stata/SE 12 and open source procedure R 4.1.1 (https://www.r-project.org/).

## Data Availability

The datasets presented in this study can be found in online repositories. The names of the repository/repositories and accession number(s) can be found below; https://www.ncbi.nlm.nih.gov/, SAMN28422338; https://www.ncbi.nlm.nih.gov/, SAMN28422339; https://www.ncbi.nlm.nih.gov/, SAMN28422340; https://www.ncbi.nlm.nih.gov/, SAMN28422341; https://www.ncbi.nlm.nih.gov/, SAMN28422342; https://www.ncbi.nlm.nih.gov/, SAMN28422343; https://www.ncbi.nlm.nih.gov/, SAMN28422344; https://www.ncbi.nlm.nih.gov/, SAMN28422345; https://www.ncbi.nlm.nih.gov/, SAMN28422346; https://www.ncbi.nlm.nih.gov/, SAMN28422347.

## Abbreviations

GPR41, forward CTT​CTT​TCT​TGG​CAA​TTA​CTG​GC; reverse CCG​AAA​TGG​TCA​GGT​TTA​GCA​A, GPR43; forward CTT​GAT​CCT​CAC​GGC​CTA​CAT, reverse CCA​GGG​TCA​GAT​TAA​GCA​GGA​G, GAPDH; forward AGT​GTT​TCC​TCG​TCC​CGT​A; reverse CGT​GAG​TGG​AGT​CAT​ACT​GG.
